# Measurement of solid size in early-stage lung adenocarcinoma by virtual 3D thin-section CT applied artificial intelligence

**DOI:** 10.1038/s41598-023-48755-5

**Published:** 2023-12-07

**Authors:** Shingo Iwano, Shinichiro Kamiya, Rintaro Ito, Akira Kudo, Yoshiro Kitamura, Keigo Nakamura, Shinji Naganawa

**Affiliations:** 1 Department of Radiology, Nagoya University Graduate School of Medicine, 65 Tsurumai-cho, Showa-ku, Nagoya, 466-8550 Japan; 2 Imaging Technology Center, Fujifilm Corporation, 2-26-30, Nishiazabu, Minato-ku, Tokyo, 106-8620 Japan

**Keywords:** Cancer imaging, Lung cancer, Tumour biomarkers, Software

## Abstract

An artificial intelligence (AI) system that reconstructs virtual 3D thin-section CT (TSCT) images from conventional CT images by applying deep learning was developed. The aim of this study was to investigate whether virtual and real TSCT could measure the solid size of early-stage lung adenocarcinoma. The pair of original thin-CT and simulated thick-CT from the training data with TSCT images (thickness, 0.5–1.0 mm) of 2700 pulmonary nodules were used to train the thin-CT generator in the generative adversarial network (GAN) framework and develop a virtual TSCT AI system. For validation, CT images of 93 stage 0–I lung adenocarcinomas were collected, and virtual TSCTs were reconstructed from conventional 5-mm thick-CT images using the AI system. Two radiologists measured and compared the solid size of tumors on conventional CT and virtual and real TSCT. The agreement between the two observers showed an almost perfect agreement on the virtual TSCT for solid size measurements (intraclass correlation coefficient = 0.967, *P* < 0.001, respectively). The virtual TSCT had a significantly stronger correlation than that of conventional CT (*P* = 0.003 and *P* = 0.001, respectively). The degree of agreement between the clinical T stage determined by virtual TSCT and the clinical T stage determined by real TSCT was excellent in both observers (k = 0.882 and k = 0.881, respectively). The AI system developed in this study was able to measure the solid size of early-stage lung adenocarcinoma on virtual TSCT as well as on real TSCT.

## Introduction

The clinical TNM classification of malignant tumors (TNM) of primary lung cancer plays an important role in therapeutic strategy decisions^1–3^. The diagnosis of the tumor (T) factor is particularly important in early-stage (0–I) lung cancer in determining the surgical procedure^4,5^. The TNM staging can be clinical (c-stage) and/or pathologic (p-stage), and the former is determined by the maximal size of the solid component (solid size) on thin-section CT (TSCT), while the latter is determined by the maximal invasive size on histopathological specimens in the eighth version of the Union for International Cancer Control (UICC) TNM classification^4,6–8^.

The solid size should be measured three-dimensionally using TSCT images with a slice thickness of 1 mm or less and multiplanar reconstruction (MPR) images^5,9,10^. However, sequential TSCT images increase the data volume and may cause problems in the transmission and storage of picture and archiving communication systems (PACS). Compared to 5 mm-thick conventional CT, 0.5–1 mm thick TSCT increases data volume by 5 × to 10 × fold. Therefore, TSCT is recommended for evaluation of solid nodules, but CT performed on 5-mm-thick sections is still common in clinical practice^11,12^. If the solid size can be measured as accurately as the TSCT on conventional 5-mm-thick CT images, the problem can be solved.

In recent years, many attempts have been made to improve the image quality of chest CT by applying artificial intelligence (AI) techniques such as deep learning (DL)^13–18^. Therefore, we attempted to generate virtual TSCT and MPR images from conventional CT images of lung cancer using DL. The purpose of this study was to investigate whether virtual TSCT can measure the solid size of early-stage lung adenocarcinoma.

## Methods

### Using AI to generate virtual TSCT

**Figure 1:**
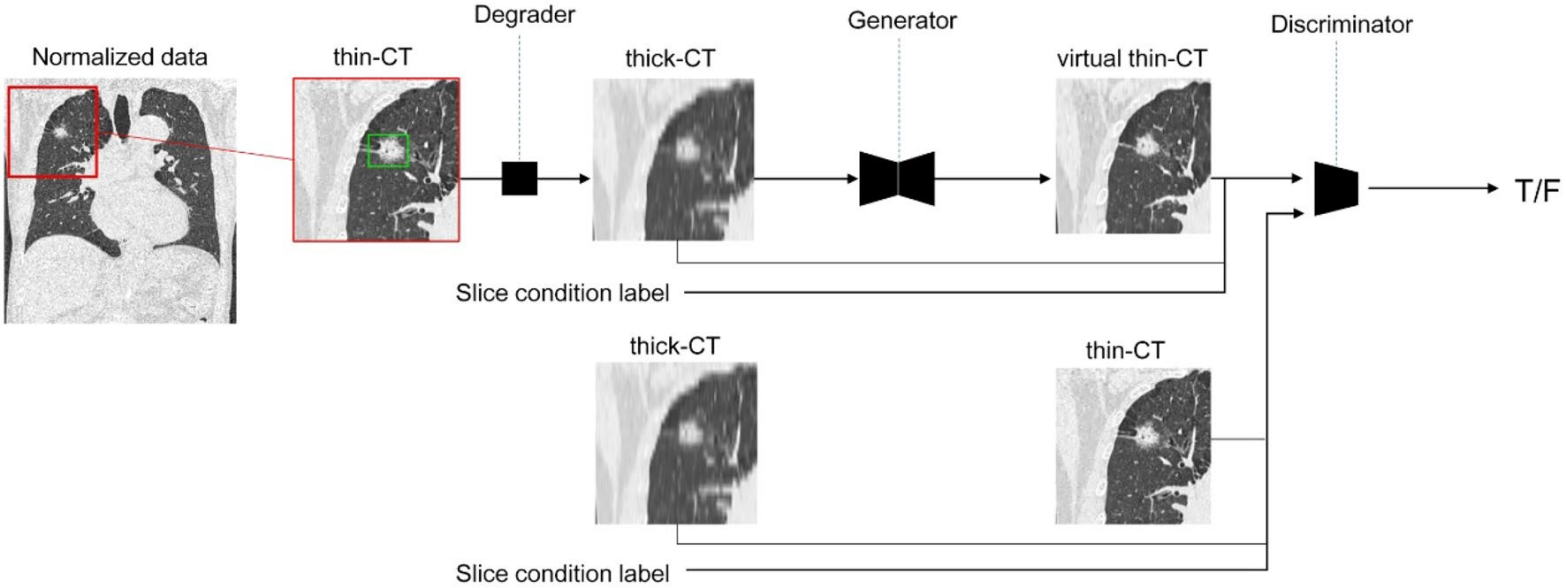
Adversarial training framework of three-dimensional (3D)-conditional generative adversarial network (GAN)-based slice interpolation. In each training iteration, patched thin-CT was randomly degraded to simulate thick-CT for the Generator input. On the other hand, for the Discriminator input, we feed generated virtual thin-CT or real thin-CT with slice condition labels associated with simulated thick-CT.

A three-dimensional (3D) conditional generative adversarial network (GAN)-based algorithm was applied to virtually generate thin-section CT from TSCT^19^. As training data, TSCT images (thickness, 0.5–1.0 mm) of 2700 pulmonary nodules prior to December 2016 were collected from the PACS of our institution. Thick-slice images with thickness/intervals of 3.0–8.0 mm were randomly simulated from the TSCT image using interpolation processing. The pair of original thin-CT and simulated thick-CT was used to train the thin-CT generator in the GAN framework, where the discriminator takes the roll to let the generator produce virtual thin-CT that is difficult to distinguish from the real thin-CT (Fig. 1). We incorporated the conditioning labels (ex. slice thickness) associated with the input CT into conditional adversarial training to improve the accuracy of the interpolation.

### Validation case collection

Using the retrieval function of PACS, we searched our institutional database for CT and pathological reports of patients with pathologically confirmed, surgically resected stage 0–I lung adenocarcinoma based on the TNM classification in the 8th version of UICC between January 2017 and March 2018. Consequently, CT images of 93 patients with early-stage lung adenocarcinoma were collected.

The clinical records of all selected cases were reviewed to obtain data on the patients’ age, sex, location of the tumor, date of preoperative CT, and surgery. Based on the pathological reports, the invasive size of the tumor was recorded, and pathological T stage (pTis, pT1mi, pT1a, pT1b, pT1c, and pT2a) was determined (Table 1).

**Table 1:** Patient and tumor characteristics.

	*n*	Mean ± SD	Range
Age (years)		66 ± 9	40–82
Male/female	34/59		
Period from CT to surgery (days)		21.7 ± 11.9	1–58
RUL/RML/RLL/LUL/LLL	31/8/21/21/12		
Solid/part-solid GGN/pure GGN	27/55/11		
Total size (mm)		19.6 ± 9.1	5–45
Solid size (mm)		11.3 ± 9.6	0–36
Clinical T stage Tis/T1mi/T1a/T1b/T1c/T2a	11/19/17/26/18/2		
Pathological total size (mm)		17.9 ± 8.7	4–42
Invasive size (mm)		13.7 ± 9.2	0–38
Pathological T stage Tis/T1mi/T1a/T1b/T1c/T2a	11/7/15/42/12/6		
In situ/lepidic/papillary/acinar/solid/mucinous	9/14/59/9/2/0		

*SD* standard deviation, *RUL* right upper lobe, *RML* right middle lobe, *RLL* right lower lobe, *LUL* left upper lobe, *LLL* left lower lobe, *GGN* ground-glass opacity.

Preoperative CT scans were performed with four types of CT scanners (SOMATOM Definition Flash, Siemens Healthcare; Aquilion, Canon Medical Systems) in the craniocaudal direction with inspiratory apnea. No contrast medium was used. The reconstructed images were 5-mm thick (conventional CT) and equal to or less than 1-mm thick (real TSCT) in the routine at our institution (Table 2).

**Table 2:** CT scan and reconstruction parameter of validation data.

Scanner	n	kVp	Conventional CT thickness (mm)	Real TSCT thickness (mm)	Kernel
Definition flash	78	120	5	0.6	I70f
Aquilion	6	120	5	1	FC52
Aquilion	9	120	5	0.5	FC52

*TSCT* thin-section CT.

**Figure 2:**
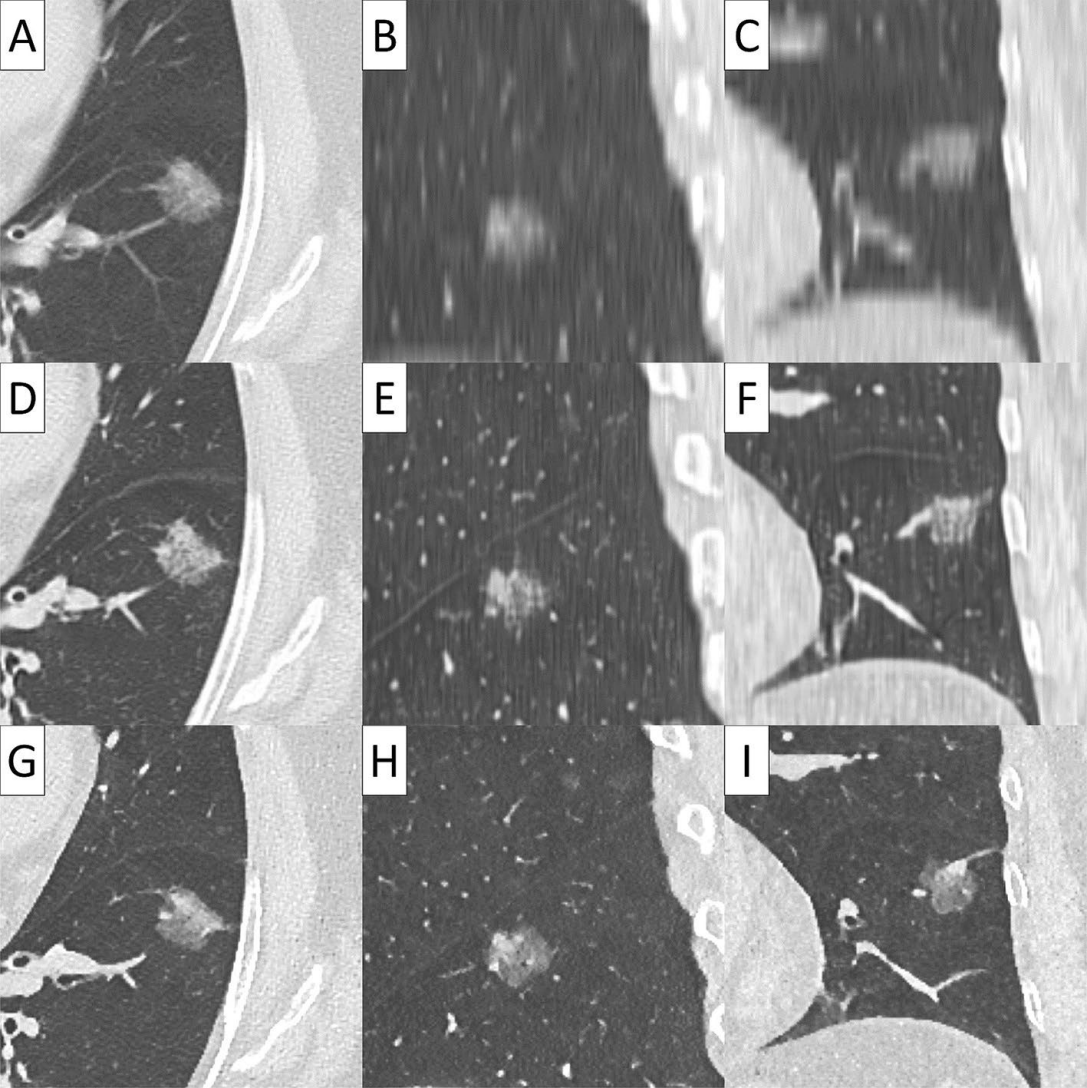
Papillary adenocarcinoma in a 72-year-old female. (**A**) Axial view, (**B**) sagittal view, and (**C**) coronal view of conventional 5-mm thick CT. (**D**) Axial view, (**E**) sagittal view, and (**F**) coronal view of virtual thin-section CT (TSCT). (**G**) Axial view, (**H**) sagittal view, and (**I**) coronal view of real TSCT.

**Figure 3:**
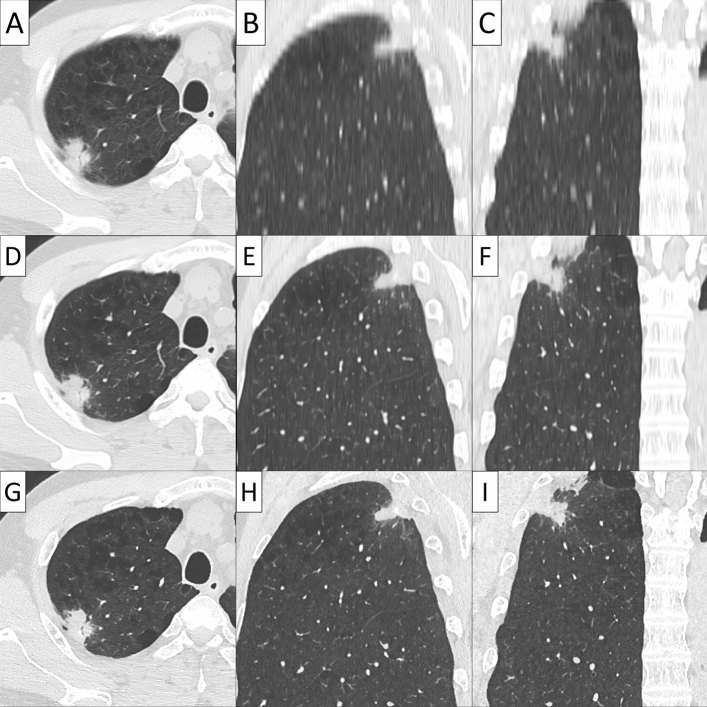
Solid adenocarcinoma in a 49-year-old male. (**A**) Axial view, (**B**) sagittal view, and (**C**) coronal view of conventional 5-mm thick CT. (**D**) Axial view, (**E**) sagittal view, and (**F**) coronal view of virtual thin-section CT (TSCT). (**G**) Axial view, (**H**) sagittal view, and (**I**) coronal view of real TSCT.

**Figure 4:**
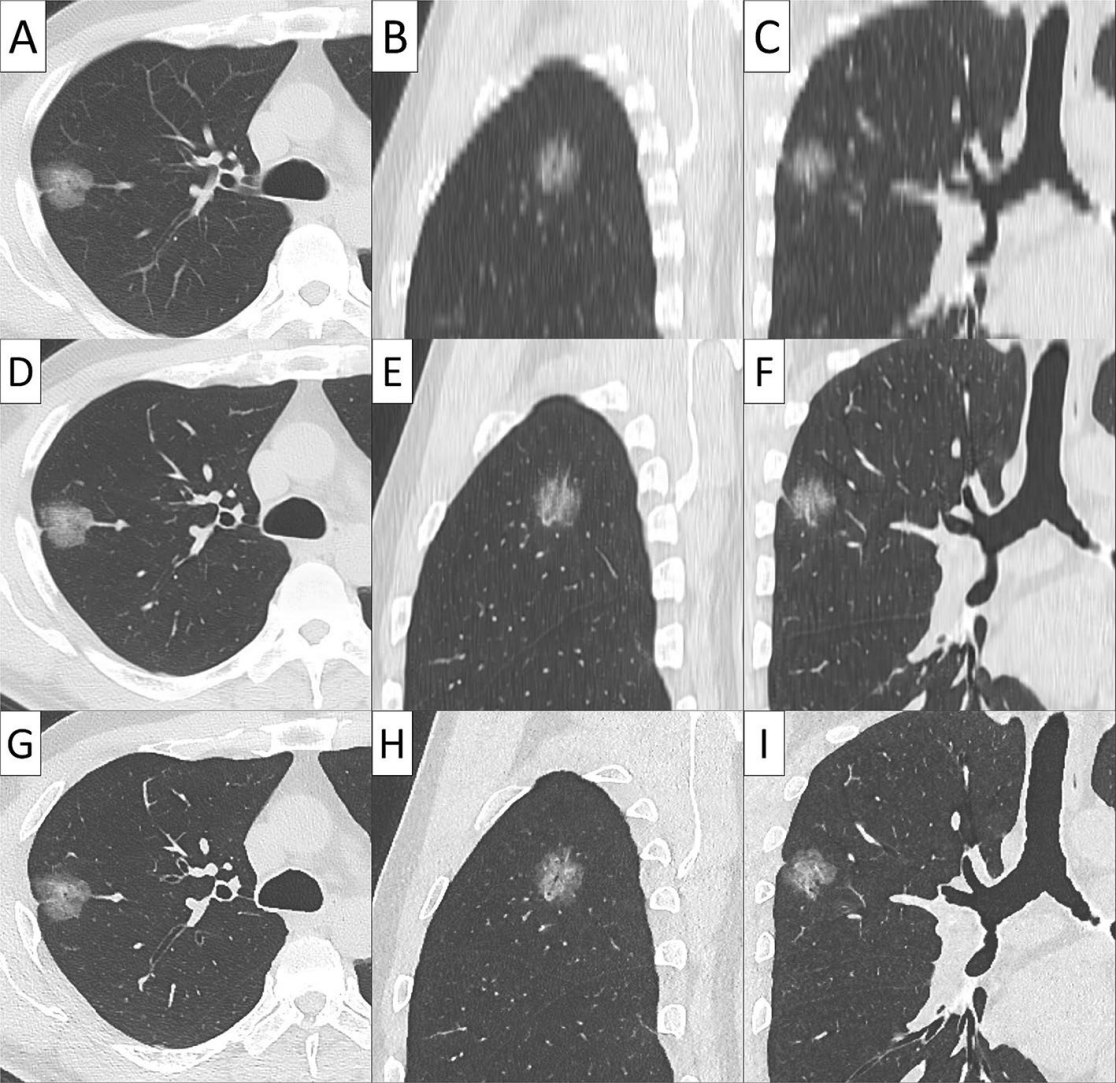
Papillary adenocarcinoma in a 47-year-old male. (**A**) Axial view, (**B**) sagittal view, and (**C**) coronal view of conventional 5-mm thick CT. (**D**) Axial view, (**E**) sagittal view, and (**F**) coronal view of virtual thin-section CT (TSCT). (**G**) Axial view, (**H**) sagittal view, and (**I**) coronal view of real TSCT.

**Figure 5:**
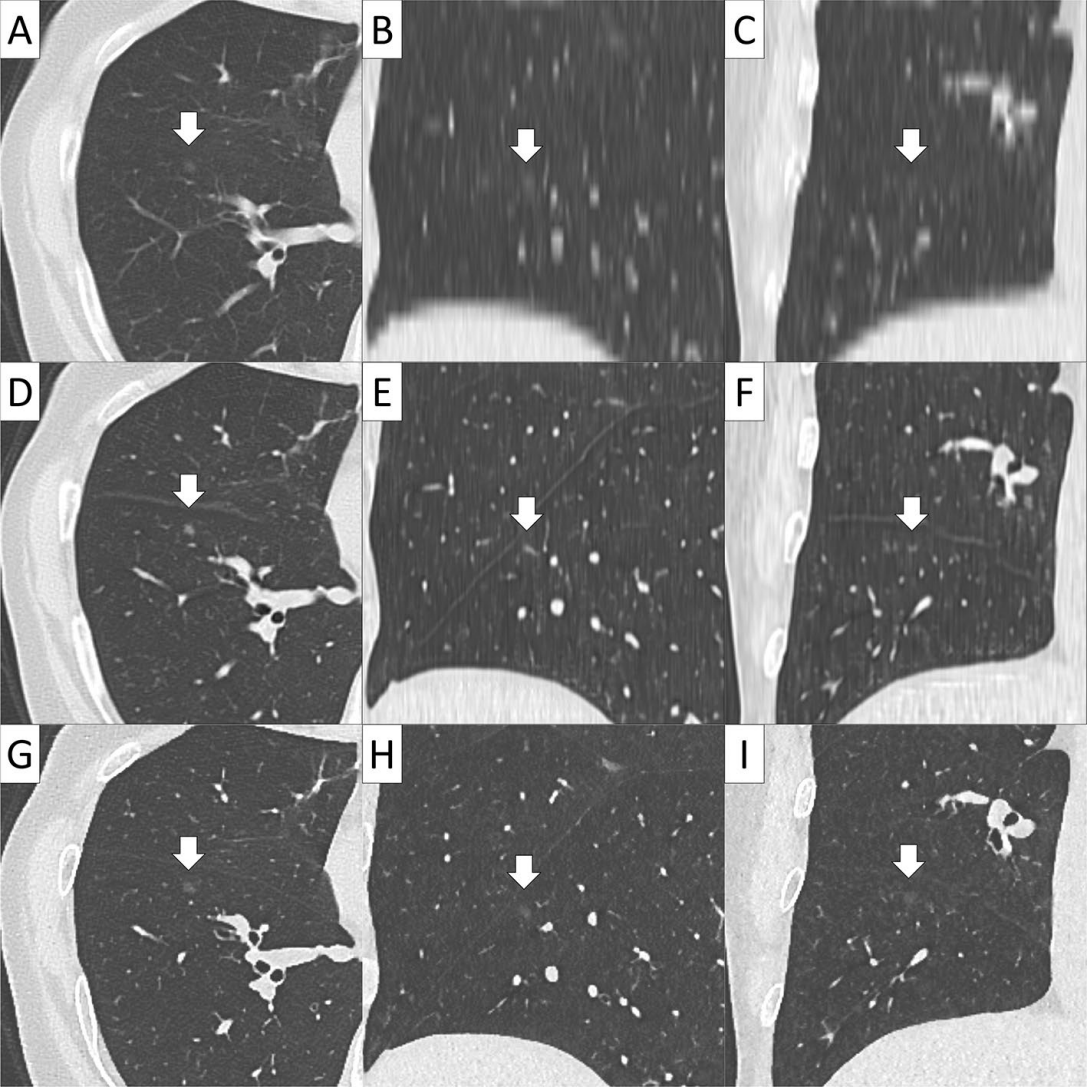
Adenocarcinoma in situ with a diameter of 5 mm in a 60-year-old female. (**A**) Axial view, (**B**) sagittal view, and (**C**) coronal view of conventional 5-mm thick CT. (**D**) Axial view, (**E**) sagittal view, and (**F**) coronal view of virtual thin-section CT (TSCT). (**G**) Axial view, (**H**) sagittal view, and (**I**) coronal view of real TSCT.

Conventional 5 mm-thick CT, virtual TSCT and real TSCT are shown in Figs. 2, 3, 4 and 5. The AI algorithm output the same results in multiple tests to the same conventional thick CT. The time required to create a 3D virtual TSCT from a conventional CT using AI was less than 5 s per case.

### Solid size measurement and clinical T staging on a 3D workstation

All reconstructed TSCT images were transferred to a 3D workstation (VINCENT V4.3, Fujifilm Medical). The CT images were displayed with window level and width settings of 1500 and − 600 HU and reviewed by two radiologists with 5 and 26 years of experience as observers A and B, respectively. Observer B was a chest radiologist who annually staged over 400 cases of lung cancer in our institution. First, two observers independently measured the maximal solid size on the MPR images (axial, coronal, and sagittal plane) obtained from virtual TSCT images. Second, they independently measured the maximal solid size on conventional CT images. In addition, observer A and B measured the maximal solid size on the MPR images obtained from the real TSCT images. To avoid memory effects, each reading experiment was carried out at intervals of at least one week.

The clinical T stage (cTis, cT1mi, cT1a, cT1b, cT1c, and cT2a) was defined by observer B based on the solid size measured by real TSCT, and this was used as the gold standard for clinical staging. In addition, each clinical T stage was determined by the solid size measured by observers A and B using virtual TSCT and conventional CT, respectively.

### Statistical analysis

First, the agreement between the two observers for solid size measurement was analyzed using the intraclass correlation coefficient (ICC). Second, the Pearson correlation coefficient was calculated with real TSCT for conventional CT and virtual TSCT, and the values were compared using the z-test for each observer. Analysis of covariance (ANCOVA) was used to compare between observers and conventional CT and virtual TSCT the solid size measurement. Third, the correspondence rates of the c-stage between the real TSCT and virtual TSCT and between the real TSCT and conventional CT were analyzed using the kappa coefficient (k). Similarly, the correspondence rate between the p- and c-stages was analyzed using k. The degree of agreement was interpreted, as follows: less than 0.20, poor agreement; 0.21–0.40, fair agreement; 0.41–0.60, moderate agreement; 0.61–0.80, good agreement; and 0.81–1.00, almost perfect agreement.

Analyses were performed using the commercial statistical software Excel 2013 (Microsoft Corporation), a statistical add-in (BellCurve version 3.20; Social Survey Research Information Corp.), and SPSS version 23 (IBM Corp.). Statistical significance was set at *P* < 0.05.

### Ethics approval and consent to participate

This retrospective study was approved by the institutional review board of Nagoya University Graduate School of Medicine, and written informed consent was waived (approval no. 2017-0471-3). The study was conducted according to the relevant guidelines and regulations.

## Results

The solid size on the conventional CT measured by observer A and B were 8.3 ± 7.9 mm and 8.4 ± 8.2 mm, respectively. The solid size on the virtual TSCT measured by observer A and B were 12.5 ± 10.0 mm and 12.4 ± 10.2 mm, respectively. The solid size on the real TSCT measured by observer A and B were 11.6 ± 9.5 mm and 11.8 ± 9.6 mm, respectively. For solid size measurements, the agreement between the two observers showed an almost perfect agreement on the conventional CT, the virtual TSCT, and real TSCT (ICC = 0.950, *P* < 0.001; ICC = 0.967, *P* < 0.001; and ICC = 0.962, *P* < 0.001, respectively). The invasive size on the postoperative pathology specimen was 13.7 ± 9.2 mm.

**Figure 6:**
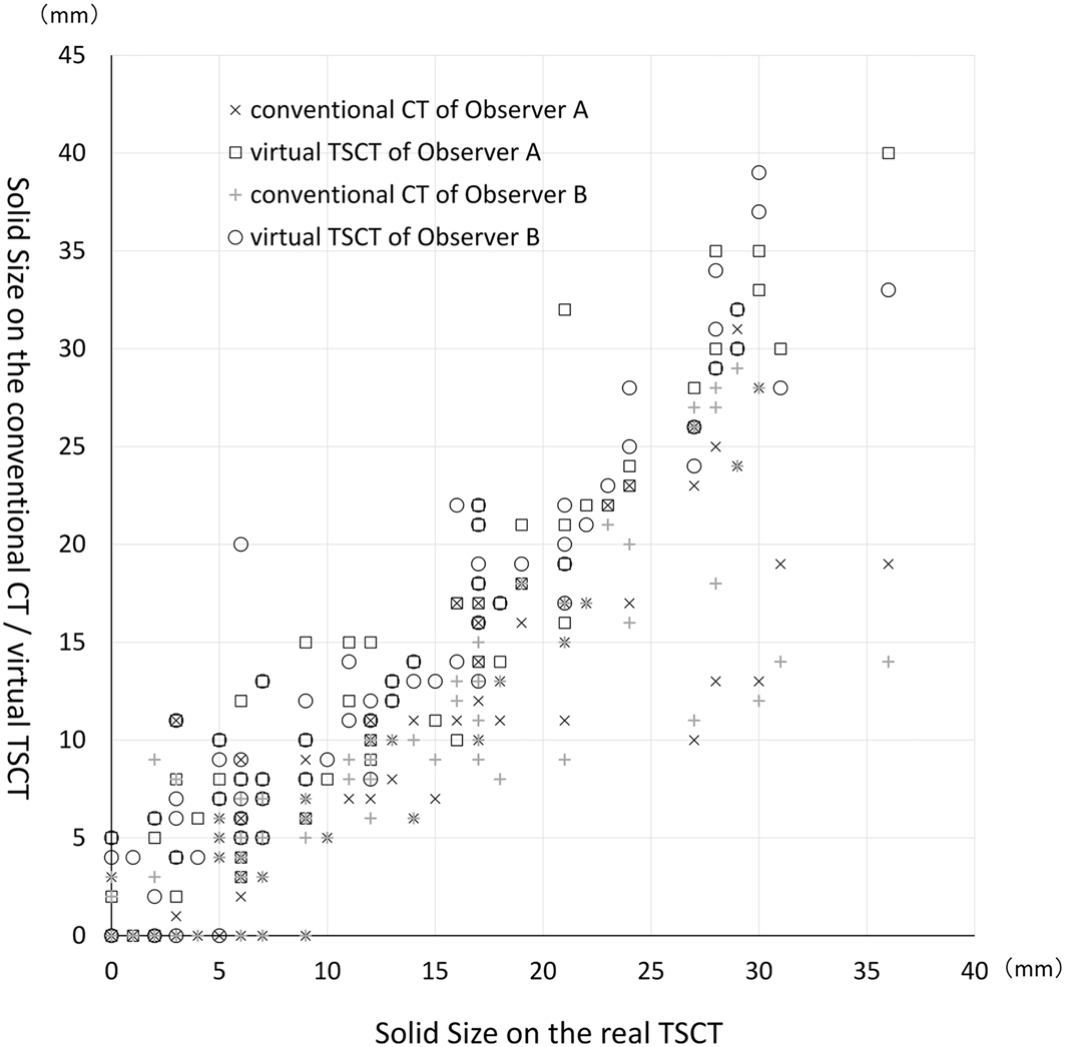
The solid size measured by observer A and B on the conventional CT and virtual thin-section CT (TSCT) plotted against the solid size measured by observer B with real TSCT.

The solid size measured on the conventional CT tended to be smaller, especially for tumors with a solid size > 2 cm (Fig. 6). The Pearson correlation coefficient with real TSCT of observer A was 0.88 for conventional CT and 0.95 for virtual TSCT, and virtual TSCT had a significantly stronger correlation than that of conventional CT (*P* = 0.003). Similarly, the Pearson correlation coefficient of observer B showed a value of 0.90 for conventional CT and 0.96 for virtual TSCT, and virtual TSCT had a significantly stronger correlation than that of conventional CT (*P* = 0.001). ANCOVA of the difference in the solid size between observers and between conventional CT and virtual TSCT revealed no significant variation between observers (*P* = 0.930), whereas a significant difference was observed between conventional CT and virtual TSCT (*P* < 0.001).

The degree of agreement between the clinical T stage determined by conventional CT and the clinical T stage determined by real TSCT was good in both observers (k = 0.757 and k = 0.780, respectively; Table 3). On the other hand, the degree of agreement between the clinical T stage determined by virtual TSCT and the clinical T stage determined by real TSCT was excellent in both observers (k = 0.882 and k = 0.881, respectively; Table 3).

**Table 3:** Comparison of clinical T stage in each observer.

	Observer A			Observer B		
	Kappa value	95% CI	P value	Kappa value	95% CI	P value
Conventional CT	0.757	0.692–0.821	< 0.001	0.780	0.716–0.844	< 0.001
Virtual TSCT	0.882	0.838–0.926	< 0.001	0.881	0.839–0.923	< 0.001

*CI* confidence interval, *TSCT* thin-section CT.

The degree of agreement between the clinical T stage determined by conventional CT and the pathological T stage determined by the invasive size of postoperative pathological specimens was moderate in both observers (k = 0.449 and k = 0.451, respectively; Table 4). In contrast, the degree of agreement between the clinical T stage determined by virtual TSCT and pathological T stage was moderate in both observers (k = 0.591 and k = 0.591, respectively; Table 4). Additionally, the degree of agreement between the clinical T stage determined by real TSCT and pathological T stage was moderate in both observers (k = 0.524 and k = 0.597, respectively; Table 4).

**Table 4:** Comparison of pathological T stage in each observer.

	Observer A			Observer B		
	Kappa value	95% CI	P value	Kappa value	95% CI	P value
Conventional CT	0.449	0.317–0.582	< 0.001	0.451	0.312–0.591	< 0.001
Virtual TSCT	0.591	0.440–0.742	< 0.001	0.591	0.418–0.765	< 0.001
Real TSCT	0.524	0.365–0.683	< 0.001	0.597	0.450–0.743	< 0.001

*CI* confidence interval, *TSCT* thin-section CT, *N/A* not available.

## Discussion

In this study, we applied AI to convert 5 mm-thick conventional CT images into virtual TSCT images and investigated whether this could accurately measure the solid size of lung adenocarcinoma. The results showed that the solid size measurements of the two observers on virtual TSCT were almost identical, and the correlation between virtual TSCT and real TSCT measurements was significantly higher than that of conventional CT. The agreement between clinical T stage determined by virtual TSCT and clinical T stage determined by real TSCT was excellent. Furthermore, the clinical T stage of virtual TSCT had a higher correlation with the pathological T stage than the T stage of conventional CT.

Virtual TSCT is an AI technique that applies deep learning to convert thick-slice CT into TSCT images. We have succeeded in synthesizing virtual TSCT images that are similar to real TSCT from thick-slice CT images by training a set of averaged 3.0–8.0 mm-thick images reconstructed from TSCT images using a number of lung nodule cases as training data. For both observers, the correlation between the measurements of virtual TSCT and real TSCT was significantly higher than that between conventional CT and real TSCT. Various factors that make measurement of the solid size of lung cancer have been reported^10,20^. For tumors larger than 2 cm, the solid size measured by conventional CT tended to be smaller. The virtual TSCT not only improves the spatial resolution of the axial section, but also improves the spatial resolution of the craniocaudal direction, which enables accurate measurement of the solid size with MPR images. Naturally, there was an excellent correlation between the clinical T stage determined by virtual TSCT and the clinical T stage determined by real TSCT.

The correlation between the clinical T stage determined by virtual TSCT and the pathological T stage was moderate but was higher than the correlation with the clinical stage determined by conventional CT. However, it was comparable to the clinical T stage determined by real TSCT. Several previous studies have reported a difference between clinical TNM stages and pathological stages^7,10,15,21^. Ahn et al. reported that the kappa values for agreement between clinical T-stage and pathological T-stage were 0.53–0.69^10^. In the present study, the kappa values for agreement between clinical T stage determined by virtual TSCT and the pathological T stage was 0.59 in both observers, which was consistent with the results of Ahn et al. Interestingly, in the article by Funai et al., clinical T-stage based on TSCT was more correlated with the postoperative recurrence than with the pathological T-stage based on the invasive size^21^. Therefore, we speculate that the clinical T-stage measured by virtual TSCT may also be correlated with prognosis. Further investigation is needed to determine whether measurement of the solid size by virtual TSCT is related to the prognosis.

Not all chest CTs are acquired with TSCT. There are several hospitals that only scan conventional CT for screening examinations. The conventional CT images obtained in the past could not be reconstructed into TSCT images. Lung nodules are often incidentally detected on conventional CT. In such cases, it is much faster to create a virtual TSCT on PACS than to reconstruct a real TSCT again from the raw CT data. In this study, we examined nodular lesions, but the virtual TSCT technique could also be applied to diffuse lung diseases. Furthermore, small pulmonary nodules are often missed on conventional thick-slice CT, but virtual TSCT is expected to improve nodule detection compared to conventional CT because of the improved 3D appearance of the nodules. We are prepared to further study the clinical usefulness of virtual TSCT.

This study had several limitations. First, it was a retrospective, single-center study. We used conventional CT images acquired from several CT systems; however, we could not try the CT systems from all manufacturers. Second, the solid size was measured manually by only two radiologists. Although the agreement between two observers showed an almost perfect agreement, to minimize bias introduced by individual difference, reading experiments with a multiple readers may have been necessary. Also, computer-aided diagnosis may be able to measure it more accurately^5^. Therefore, we plan to improve the software to measure the solid size and volume automatically from virtual TSCT. Finally, we only focused on the solid size of lung cancers and did not examine the edge characteristics, such as spiculation. The AI algorithm should improve upon this part by increasing the training data. The virtual TSCT still requires ongoing refinement. The error range of the virtual TSCT can extend up to 9 mm, potentially leading to unnecessary surgeries. The integration of AI in medicine can sometimes result in unforeseen risks.

In conclusion, we developed an AI system that reconstructs virtual TSCT images from conventional CT images by applying DL. It was possible to measure the solid size of early-stage lung adenocarcinoma on virtual TSCT as well as on real TSCT, which could be useful for the clinical TNM staging of lung cancer.

## Data Availability

The datasets used and/or analyzed during the current study are available from the corresponding author on reasonable request.

## Abbreviations

TNM: TNM classification of malignant tumors
UICC: Union for International Cancer Control
TSCT: Thin-section CT
MPR: Mutiplanar reconstruction
PACS: Picture and archiving communication systems
AI: Artificial intelligence
DL: Deep learning
3D: Three-dimensional
GAN: Generative adversarial network
ICC: Intraclass correlation coefficient
k: Kappa coefficient
